# More than words: Speech production in first-episode psychosis predicts later social and vocational functioning

**DOI:** 10.3389/fpsyt.2023.1144281

**Published:** 2023-04-14

**Authors:** Michael Mackinley, Roberto Limongi, Angélica María Silva, Julie Richard, Priya Subramanian, Hooman Ganjavi, Lena Palaniyappan

**Affiliations:** ^1^Robarts Research Institute, University of Western Ontario, London, ON, Canada; ^2^Lawson Health Research Institute, London, ON, Canada; ^3^Department of Psychiatry, University of Western Ontario, London, ON, Canada; ^4^Department of Medical Biophysics, Western University, London, ON, Canada; ^5^Department of Psychiatry, Douglas Mental Health University Institute, McGill University, Montreal, QC, Canada

**Keywords:** language, thought disorder, First Episode Psychosis, schizophrenia, NEET (neither education employment or training)

## Abstract

**Background:**

Several disturbances in speech are present in psychosis; however, the relationship between these disturbances during the first-episode of psychosis (FEP) and later vocational functioning is unclear. Demonstrating this relationship is critical if we expect speech and communication deficits to emerge as targets for early intervention.

**Method:**

We analyzed three 1-min speech samples using automated speech analysis and Bayes networks in an antipsychotic-naive sample of 39 FEP patients and followed them longitudinally to determine their vocational status (engaged or not engaged in employment education or training—EET vs. NEET) after 6–12 months of treatment. Five baseline linguistic variables with prior evidence of clinical relevance (total and acausal connectives use, pronoun use, analytic thinking, and total words uttered in a limited period) were included in a Bayes network along with follow-up NEET status and Social and Occupational Functioning Assessment Scale (SOFAS) scores to determine dependencies among these variables. We also included clinical (Positive and Negative Syndrome Scale 8-item version (PANSS-8)), social (parental socioeconomic status), and cognitive features (processing speed) at the time of presentation as covariates.

**Results:**

The Bayes network revealed that only total words spoken at the baseline assessment were directly associated with later NEET status and had an indirect association with SOFAS, with a second set of dependencies emerging among the remaining linguistic variables. The primary (speech-only) model outperformed models including parental socioeconomic status, processing speed or both as latent variables.

**Conclusion:**

Impoverished speech, even at subclinical levels, may hold prognostic value for functional outcomes and warrant consideration when providing measurement based care for first-episode psychosis.

## 1. Introduction

Schizophrenia is an illness of disordered thought, with symptoms often reflected in disturbances in language and communication (1). An impairment of verbal communication is one of several diagnostic features of schizophrenia, with a strong posited genetic component (2), but not all patients with schizophrenia exhibit clinically identifiable disordered speech. Speech disturbances, referred to as formal thought disorders (FTD), can be classified into positive or negative FTD. Positive FTD includes phenomena such as derailment, tangentiality, or in more severe cases, neologisms or even complete incoherence (schizophasia). Alternatively, negative FTD captures the characteristic poverty of speech that many patients experience (1). While several scales have been developed with the goal of identifying these elements of speech, such as the scale for Thought, Language, and Communication (TLC) (3) or the Thought Language Index (TLI) (4), many of the speech disturbances in psychosis are too subtle to be captured by clinicians during a cross-sectional clinical interaction (5).

Recent work has focused on identifying subtler forms of speech variation in naturalistic speech among schizophrenia samples, a goal that has been aided by the proliferation of automated linguistic analysis tools (6, 7). The utilization of these automated speech analysis software programs allows complex analysis of speech without the burdens (and expense) of manual scoring. Automated linguistic analyses have allowed researchers to identify disturbances in multiple levels of speech in schizophrenia, from phonological, morphological, syntactic, and pragmatic levels (8), and have been utilized in predicting psychosis onset in at risk populations (9).

While it is intuitive that social and vocational outcomes may relate to one’s verbal abilities, the body of research demonstrating this link in schizophrenia have several limitations that preclude the use of linguistic features in functional prognostication. First, much of this work has been based on language impairments in experimental, rather than naturalistic, paradigms where the semantic space is defined by the researcher (10) [e.g., using verbal fluency tests (11, 12)]. Secondly, even in studies assessing unconstrained speech, objective aspects of conversations are not considered; instead, the clinically judged construct of thought disorder is employed. While studies have observed associations between functional outcomes and negative FTD (specifically poverty of speech and content) (13, 14), other studies have reported that positive, but not negative, elements of FTD are related to functional outcome (15). These inconsistencies in the extant literature may be related to the difficulties surrounding the clinical assessment of formal thought disorder. Thirdly, most studies to date make cross-sectional correlations between functioning and verbal assessments; there is a notable lack of longitudinal data to clarify whether the verbal deficits temporally precede (and thus lie on the causal pathway of) poor functioning seen in schizophrenia. Furthermore, functional outcomes in many prior studies have been conflated with severity of psychopathology when using tools such as Global Assessment of Functioning (15), and a lack of satisfactory definition of social dysfunction (16, 17). In addition, exposure to antipsychotics over a long period of time alters the nature of speech and our ability to assess FTD (18), thus necessitating the study of minimally treated or drug-naive subjects. Demonstrating this relationship will be of critical value in improving clinical decisions during early intervention based on long-term prognostic outlook, which at present is challenging to assess. To address this crucial gap, we sought to identify linguistic features of speech in an untreated FEP sample using a computational linguistic approach called parts-of-speech tagging implemented through Cohmetrix (19), and the Linguistic Inquiry Word Count (LIWC) (20).

While the feature space for selecting linguistic variables in relation to functional outcomes is relatively large, we focus exclusively on the variables that we have previously studied in an overlapping sample and demonstrated to have clinical relevance. In a prior cross-sectional analysis on this sample of untreated subjects, Mackinley et al. (21) used Coh-Metrix automated speech analysis software (19) to compare FEP patients and healthy controls on a number of variables at the word, sentence, and higher-order level. In this study, patients showed reduced speech production (number of words) and higher pronoun use compared to their healthy control counterparts but did not differ in a variety of other higher-order linguistic metrics (narrativity, formality, referential cohesion, or deep cohesion). Five types of connectives were analyzed in this earlier study including: causal connectives (words used to connect a cause to an effect), logical connectives (words linking two logically connected elements), temporal connectives (words to put ideas in order of time), contrastive connectives (words to compare and contrast ideas), and additive connectives (words used to add information, e.g., “additionally,” “moreover”). The use was analyzed using data driven principal factor analysis, two factors, and one with a positive loading on “all connective types” and the second “acausal temporal connective factor” reflecting reduced use of causal and contrastive connectives, but higher use of temporal linkages and additive connections appeared. While patients and healthy controls employed these connective factors in a comparable manner during the picture description tasks, patients with higher connectives use had higher scores on clinically rated conceptual disorganization (21). This suggests that aberrant linguistic connective use may contribute to the clinician’s detection of disorganized thought.

In an overlapping cross-sectional sample, we (22) analyzed the picture description speech samples using the Linguistic Inquiry Word Count (LIWC) software package (20) to determine the relative proportion of content words and function words. From this parts-of-speech tagging, we determined Pennenbaker’s Analytic Thinking scores (higher scores suggesting a well-formed hierarchical thinking style suitable for academic expressions, and lower scores suggesting a narrative style which is more intuitive and episodic in nature) (23). A higher analytic score (more categorical thinking style) is linked with academic success due to this linguistic style’s use in academic and professional settings (23). We observed that compared to HC, patients showed reduced analytic thinking in their speech. Further, among FEPs, reduced analytic thinking related to higher clinical metrics of disorganization (22). This suggests that less structured, less content-based speech may contribute to the clinician’s detection of disorganized thought. Thus, it is possible that among FEP patients, analytic thinking styles are associated with later academic and occupational success; however, little evidence to assess this question has been gathered.

With longitudinal functional outcome data from this cohort, we aim to ascertain the role of connectives, analytic thinking index, total number of words, and frequency of pronouns on vocational status and social and occupational functioning ascertained after 6-to-12 months of treatment in an early intervention setting. The selected linguistic variables tap on distinct aspects of message generation and grammatical encoding in Bock and Levelt’s language processing model (24). At the generation level, total number of words (verbosity) relates to the production plan (25); at the functional level, lexical selection influences the frequency of pronouns, while positional processing involving the assembly of constituent words influences the connective use and analytic thinking index. Given the prior observations that “negative FTD” relates more strongly to functional outcomes than “positive FTD,” we expected a reduction in total number of words used during a picture description will be predictive of later functional outcomes. To this end, we used a Bayes network (a directed acyclic graph) to (1) identify dependencies among the baseline linguistic variables and vocational status or social functioning after six to 12 months of treatment in an early intervention program for psychoses and (2) parameterize these dependencies in terms of conditional probability distributions. In the network, the dependencies are represented as connections (edges) between nodes (variables) identified through a prototypical constraint-based algorithm (26, 27). Parameters (conditional probability distributions) are found *via* maximum likelihood estimation (28). We assessed the contribution of other explanatory variables such as parental socioeconomic status and speed of cognitive processing using probabilistic models of functional outcome. We quantified social functioning using the widely used Social and Occupational Functioning Assessment Scale (SOFAS) as a continuous measure, and a macroeconomic indicator of productivity in young adults reflecting participation in active Employment Education or Training (EET vs. not-EET or NEET) status as a categorical measure, as employed in our previous brain imaging study (29).

## 2. Method

### 2.1. Participants

Data were collected from 39 treatment naïve FEP patients recruited from the Prevention and Early Intervention Program for Psychoses in London, Ontario, Canada, as reported in a previously published manuscript (21). All participants were in the acute phase of the illness, with fewer than 2 weeks of antipsychotic exposure lifetime. The mean lifetime defined daily dose was *M* = 2.31, SD = 3.68, with *n* = 14 being completely drug-naive (36%). Over the subsequent year, patients were longitudinally followed with assessments of social and occupational functioning completed when clinically stable between 6 and 12 months following the initial assessment. All participants used in the present analysis were native English speakers.

### 2.2. Clinical and linguistic assessment procedure

The local Research Ethics Board (Western University) approved all study procedures, and all patients provided informed consent before participating. All patients were enrolled in a first-episode psychosis program over the next 12 months, and we ascertained their social and vocational status between 6 to 12 months after entering treatment. Due to the need for multiple information sources, not all patient follow-ups were assessed at precisely the same time point after the onset of illness.

Licensed psychiatrists conducted all clinical interviews and rating scales to determine illness severity, and rule out exclusionary diagnoses (substance abuse, neurologic disorders). Graduate-level research assistants completed cognitive assessments and the Thought Language Index (TLI) interview and rating. During the TLI procedure, three 1-min speech samples were induced in response to photographs from the Thematic Apperception Task (30). Scorers of TLI interview were blinded to participant status consistent with the procedure described by Sommer et al. (31).

The Positive and Negative Syndrome Scale-8 Item (PANSS-8), which is highly correlated with the full 30-items PANSS (32), was utilized to measure the severity of clinical symptoms. Functional assessments were based on multiple sources of information (patient interviews, information from the psychiatrist providing care, case managers, and when required information from family members). Measures of social and occupational functioning were assessed using the Social and Occupational Functioning Assessment Scale (SOFAS) (33) at baseline and follow-up. The SOFAS is a single-item measure of functioning scored between 1 (indicating a persistent inability to maintain minimum even basic function) and 100 (superior functioning in a wide range of activities). In our study, SOFAS scores considered current functioning (rather than the highest level of functioning over the past year). Vocational assessments were conducted using a binary NEET status (not in employment education or training). Patients were deemed to be NEET (vocationally inactive) if they were unemployed and not in any form of schooling/education for more than half of the time since the onset of treatment for psychosis. Individuals classified as EET were engaged in work or school for more than half of the duration of treatment (vocationally active). This definition considers a longer period than the 1-week period used by the Organization for Economic Co-Operation and Development (OECD) (34), but is consistent with its use in early intervention services for psychosis (35, 36). When inconsistencies between patient and care provider accounts were noted, a consensus was reached among the members of the research team.

### 2.3. Instruments

#### 2.3.1. Linguistic inquiry word count

Linguistic Inquiry Word Count Software (LIWC 2015 Edition) uses a computational-lexical approach, which provides summaries of psycholinguistic dimensions (i.e., analytic thinking score) and pre-defined content word themes (e.g., negative emotion words) derived from psychometric rates. In the two-step process, LIWC analyzes the current target word contained in texts comparing and matching every single word against master dictionaries using its own language corpora composed of “almost 6,400 words, word steams, and selected emoticons from a sample of ~181,000 text files.” Secondly, a standard LIWC computes the percentage of co-occurrences. LIWC has recently gained attention in several research areas establishing the relationship between linguistic-thinking styles and both personality traits, and mental health conditions.

#### 2.3.2. Coh-Metrix 3.0

Coh-Metrix (37) is a web-based automated speech analysis software that computes basic and higher-level linguistic variables from written and spoken speech samples. The software automatically computes several lower order (e.g., word counts, frequency of pronoun use, and use of connectives) and higher-order (e.g., narrativity, cohesion, and text formality) linguistic variables (19). While initially implemented for the analysis of larger text segments, the software has been applied in the analysis of brief language samples in clinical populations previously (38). Though there are no requirements for minimum number of words for applying Coh-Metrix to study texts, analyses of readability and cohesion have been generally reported for written materials with 100 words or above (39, 40). The incidence scores are based on frequency of occurrence of different parts of speech (e.g., pronouns, connectives etc.) in the units of numbers per 1,000 words. We based our project on the work in Willits et al. (41) with the focus of Coh-Metrix output on the frequency of connectives use as described in MacKinley et al. (21).

### 2.4. Statistical (Bayesian) analyses

For descriptive analyses, we used the JASP software (JASP version 0.16.3, 2022) to report Bayes factors against the null model (BF_10_). Briefly, if BF_10_ < 2, we accepted the null hypothesis, whereas if BF > 2 provides support for the alternative hypothesis. To answer the research question, we used a prototypical constraint-based algorithm (PC) (26, 27) within the context of a Bayes network (a probabilistic graphical model) to identify dependencies in a set of variables. This set comprised NEET (6–12 months), SOFAS score (6–12 months), total words spoken, analytic thinking score, all connectives score, acausal connectives score, and pronoun use (all at baseline). We also included PANSS-8 total score as a nuisance variable to control disease severity at the time of linguistic data collection. The algorithm yielded a Bayes network upon which we applied an expectation maximization algorithm (42) to perform maximum likelihood estimation of parameters (parameters learning). Finally, we made a series of inferences (conditional probability queries in terms of causal and evidential reasoning) aiming to explain the relationships between our variables of interests (total words spoken, analytic thinking, connectives use, and pronoun use).

## 3. Results

### 3.1. Descriptive statistics

When baseline characteristics of patients who went on to be vocationally active (EET) were compared to patients that went on to be vocationally inactive (NEET), no evidence for group differences were seen for medication exposure, duration of untreated psychosis, age, sex, parental Socioeconomic status (SES), or the use of cannabis, alcohol, or tobacco, or symptom severity at baseline. As expected, given the overlapping nature of the SOFAS scale and vocational activity, very strong support was found that NEET patients differed from EET patients in measures of follow-up SOFAS score (BF_10_ = 55.50; EET mean = 65.00, SD = 10.54; NEET mean = 46.47, SD =18.28). EET patients produced an average of 18% more speech in the three 1-min TLI interview trials than their NEET counterparts, providing support that patients who speak more words at baseline would go on to be vocationally active (BF_10_ = 2.42; EET mean = 123.22, SD = 38.50; NEET mean = 104.40, SD = 24.19). Finally, we report moderate evidence that those that perform better on the Digit Symbol Substitution Test (DSST), a measure of processing speed, would go on to be vocationally active (BF_10_ = 3.32; EET mean = 57.87, SD = 14.72; NEET mean = 46.92, SD = 12.14). We report no differences on other linguistic variables of interest (Table 1).

**Table 1:** Demographic and linguistic characteristics of sample.

Variable	All patients *n* = 39	Patients not in education employment or training (NEET) *n* = 18	Patients engaged in employment education or training (EET) *n* = 21	BF_10_	95% highest density interval
*Demographic and clinical variables*					
Sex (Male/Female)	32/7	16/2	16/5	1.00	−1.72, 0.77
Age [M (sd)]	22.53 (4.76)	23.58 (6.02)	21.58 (3.15)	0.60	−0.955, 0.25
NS-SEC [M (sd)]	3.76 (1.20)	4.28 (1.07)	3.25 (1.02)	1.11	−1.14, 0.12
DUP in months [M (sd)]	8.82 (11.86)	7.57 (8.21)	10.00 (14.67)	0.32	−0.58, 0.54
Defined daily doses [M (sd)]	2.31 (3.68)	2.39 (3.65)	2.27 (3.80)	0.36	−0.53, 0.59
Non-antipsychotic meds (Y/N)	9/30	3/15	6/15	1.16	−1.59, 0.77
Tobacco smoker (Yes/No)	11/25	7/11	5/16	1.00	−1.61, 0.80
CAST score [M (sd)]	13.5 (6.59)	15.13 (6.65)	11.87 (6.34)	0.35	−1.07, 0.25
AUDIT-C [M (sd)]	2.64 (3.12)	2.07 (2.22)	3.31 (3.90)	0.53	−0.34, 0.99
PANSS-8 total [M (sd)]	26.46 (7.21)	27.44 (6.65)	25.62 (7.71)	0.40	−0.79, 0.36
PANSS-8 positive [M (sd)]	13.08 (2.98)	13.44 (2.72)	12.74 (3.24)	0.39	−0.77, 0.38
PANSS-8 negative [M (sd)]	7.76 (4.46)	8.33(4.52)	7.21 (4.44)	0.40	−0.79,0.37
CGI-severity [M (sd)]	5.34 (1.09)	5.44 (0.86)	5.26 (1.28)	0.35	−0.71, 0.44
SOFAS [M (sd)]	38.31 (12.50)	34.40 (8.35)	41.10 (14.32)	1.27	−0.10, 1.16
SOFAS 6–12 month [M (sd)]	55.74 (17.46)	46.47 (18.28)	65.00 (10.54)	55.50	0.39, 1.81
DSST [M (sd)]	52.40 (14.42)	46.92 (12.14)	57.87 (14.72)	3.32	0.06, 1.34
Months to NEET assessment [M (sd)]	7.95 (2.89)	8.17 (3.38)	7.75 (2.41)	0.34	−0.69, 0.45
*Linguistic variables of interest*					
Total words spoken	115.08 (34.03)	104.40 (24.19)	123.22 (38.5)	2.42	0.01, 1.29
Analytic thinking score	55.27 (21.65)	57.94 (22.24)	53.25 (21.52)	0.35	−0.69, 0.45
All connectives	0.095 (1.16)	−0.08 (1.61)	0.22 (1.17)	0.33	−0.50, 0.64
Acausal connectives	0.128 (0.99)	0.08 (0.79)	0.17 (1.14)	0.38	−0.39, 0.76
Pronoun use/thousand words	107.17 (24.11)	105.86 (19.93)	108.17 (27.83)	0.33	−0.50, 0.64

M, Mean; SD, standard deviation; NS-SEC, National Statistics -socioeconomic classification; DUP, Duration of Untreated Psychosis; CAST, cannabis abuse screening test; AUDIT-C, Alcohol use disorders identification test; PANSS, Positive and Negative Syndrome Scale – 8 Item Scale; CGI-Severity, Clinical Global Impression – Severity; SOFAS, Social and Occupational Functioning Assessment Score; DSST, Digit Symbol Substitution Test; BF, Bayes Factor.

### 3.2. Bayesian network analyses

**Figure 1:**
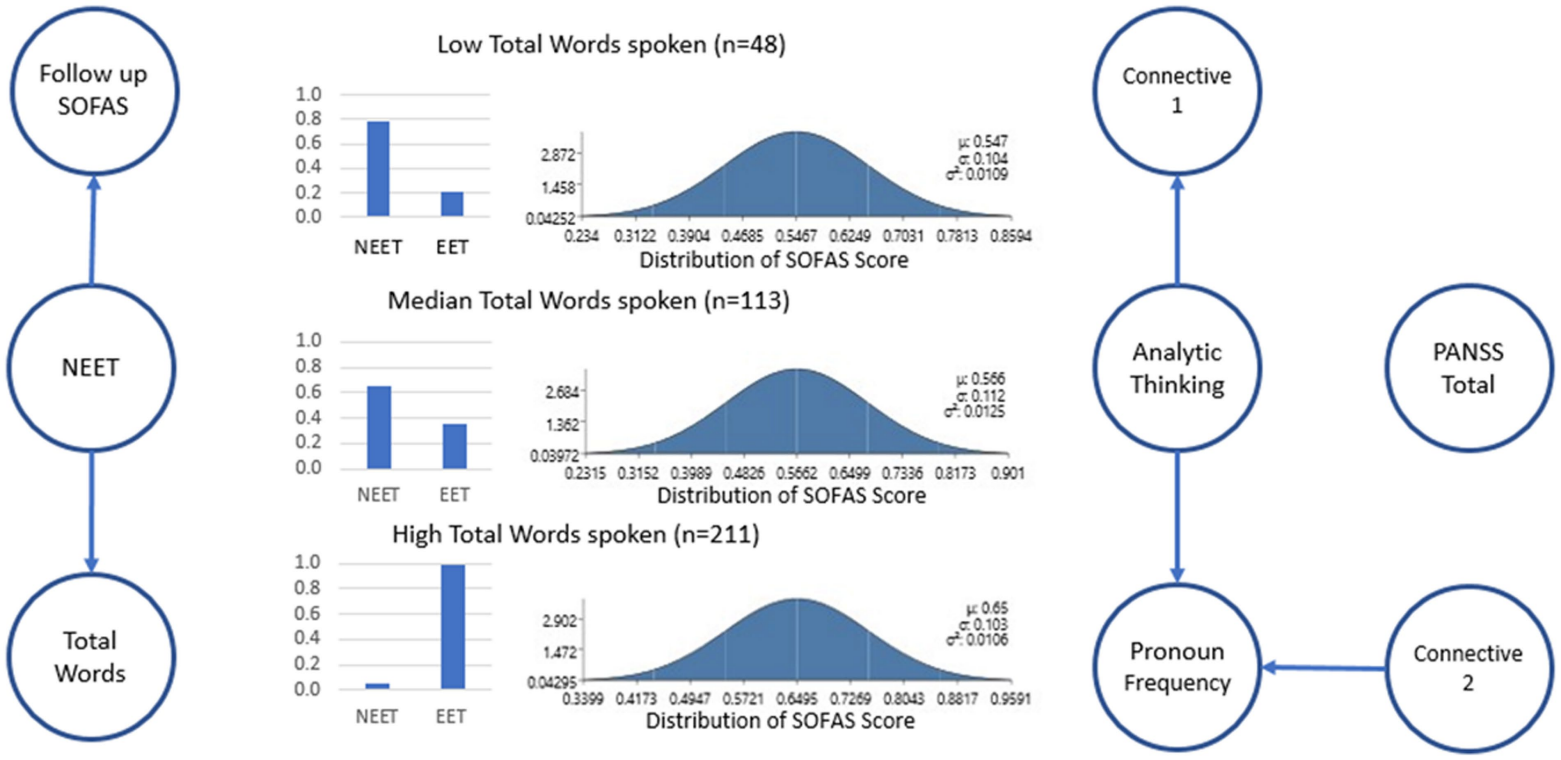
Bayes network that resulted from the application of the PC algorithm. Only the total number of words directly relates to NEET and indirectly relates to SOFAS. Furthermore, whereas the PANSS score was independent of all the other variables, the remaining predictors (Analytic thinking, Total connectives, acausal connectives, and pronoun use) show conditional dependencies among themselves. Binarized NEET/EET Probability and distribution of predicted SOFAS scores are shown based on Low, Median, and High number of words spoken. We have modified the figure to better explain the structure of the Bayes network by indicating that “circles represent variables of interest.” Arrows (i.e., directed edges) connecting circles indicate a causal influence from the ancestor to the descendent—parameterized in a conditional probability distribution. For example, a line showing follow-up SOFAS score is dependent upon NEET status. Note that there are no edges between “total PANSS” and the other variables, indicating independence. *SOFAS, Social and Occupational Functioning Assessment Scale; Connectives 1, All connectives use; Connectives 2, Acausal Connectives use; PANSS, Positive and Negative Syndrome Scale 8-Item Version; NEET, Not in Employment, Education or Training; EET, Engaged in employment education, or training.

While a causal network (indicated by the directionality of the arrows in the graph, Figure 1) was observed among the linguistic variables of interest (the two connectives factors, pronoun use, and analytic thinking style), the graphical probabilistic model revealed that only the total number of words showed a direct association with NEET and an indirect association with SOFAS (Figure 1). The expectation maximization algorithm converged (Log likelihood = −712.39). We further investigated whether this model better explained the data than a null model. To this end, we applied the expectation maximization algorithm to a model without the direct and indirect causal relationships identified above and used the Bayesian information criterion (BIC) number two adjudicate between models. We confirmed that the converged null model (Log likelihood = −723.28, BIC = 1,512) underperformed the model estimated *via* the PC algorithm (BIC = 1,504).

However, the number of words one employs during a descriptive task may vary based on factors such as social environment during early development (specifically parental SES) (43) and cognitive capacity indexed by processing speed (44) both of which may also affect the later vocational outcomes. To address this, we undertook a specific model comparison approach with self-reported parental socioeconomic status and digit symbol substitution score (a proxy for processing speed) added into our model with four contingencies and compared using the BIC numbers. The first model (M1) comprised total words conditioned upon both the DSST and SES. In the second model (M2), total words were conditional on only DSST. In the third model, total words were conditional on SES. Finally, in model 4 (M4) neither DSST nor SES influence the total number of words. The model comparison procedure yielded M4 as the best model (BIC_M1_ = 1777, BIC_M2_ = 1770, BIC_M3_ = 1774, BIC_M4_ = 1767). This indicates that despite the putative role of processing speed and SES in vocational outcomes among patients, the role of reduced speech production is best considered as an independent predictor.

Directionalities (i.e., causality) in the graph (Figure 1) indicate that both the total number of words and the SOFAS score explain the NEET score. Interestingly, once the NEET score is known the number of words and SOFAS scores are independent of each other. In consequence, the directionalities in the graph allow us to estimate the probability distribution of NEET and SOFAS given an observed total number of words (conjointly). For example, for a patient that produces 48 words on average, the probability of NEET is 79.8%. On the other extreme, if the patient produced 211 words, they would have a probability of EET (i.e., NEET = 0) with 99% chance. Finally, at the midpoint of the observed distribution of word count, a patient in the 50th percentile (median = 113) would have 64.8% chance of being in the NEET category. Similarly, with 48 words spoken we could estimate the follow-up SOFAS with a distribution of (*m* = 55, sd = 10.9). With a median number of words spoken (113 words), we would expect a similar score (*m* = 56, SD =11). However, improvements in follow-up SOFAS scores can be seen in individuals with high speech production (211 words spoken) could expect an elevated SOFAS score (*m* = 65, SD =10) a 10-point difference from their peers.

Finally, to test if a constrained word production exercise (semantic fluency task) carries the same predictive value for vocational success as word production during our conversational task with a referent (picture description), we undertook further analysis. In a subgroup of patients (*n* = 22), we gathered data from the Category Fluency test (animals), but noted that baseline category fluency (number of correct items) did not differ notably between EET and NEET patients (BF_10_ = 1.163; EET mean = 19.69, SD = 4.36; NEET mean = 16.33, SD = 4.27). This suggests that word generation during naturalistic speech has a specific prognostic value in predicting social and vocational outcomes in first-episode psychosis.

## 4. Discussion

This study sheds light on how the way we speak when experiencing acute psychosis may provide insight into our occupational/functional outcomes in the first year of early intervention. We report three major findings: (1) Speech production (total number of words spoken) during a three-minute descriptive task at the time of first presentation with psychosis, explained significant variance in NEET status after 6–12 months of treatment; (2) measures of parental socioeconomic status and processing speed did not explain this relationship; and (3) the linguistic features included in our analysis (connectives, pronoun use, and analytic thinking scores) formed their own causal network (i.e., inter-related) but were not related to vocational or social outcomes. Thus, the ability to find a productive vocational status following the experience of psychosis relates to the number of words an individual manages to deploy during a discursive task of describing a picture to another person, irrespective of parental social background, one’s personal speed of processing information and linguistic style of expression, and the severity of core symptoms (PANSS-8 total). These findings supply an objectively detectable and intuitive speech metric that requires no clinical judgment as a prognostic marker of functional outcome. This takes rater-related factors out of consideration when considering prognosis, potentially complementing clinical decisions that may require an assessment of longer-term outcomes (e.g., duration of case management, employment, and placement support).

Individuals with robust speech production had a “protective” effect with respect to functional deterioration. While those with median speech production (113 words) still had an above chance level of poor vocational outcomes (65% NEET), the effects of high speech production on vocational outcomes were far more positive; our modeling would predict that patients with speech production on the upper tail of the distribution (211 words) to have a 99% chance of being vocationally active. There are several hypotheses that could explain this association between the abundance of speech production with good vocational outcome. First, patients with high speech production are far less likely to have broader dysfunction in other negative domains. Poverty of speech has been consistently associated with affective flattening (45) and reduced symptom remission in negative domains (46), as well as likely a marker for underlying cognitive deficits (47). While this contributes a strong case for why lower speech production is likely to impair vocational prospects, it fails to make an affirmative case for good outcomes among those producing higher speech. It is likely that the benefit of speech production to good vocational prospects related to patients with more speech production being rated as more socially adept and desirable by peers and employers. In both healthy control and patient samples, social skills are highly correlated with gaining and retaining competitive employment (48). Among the patient population, the social threshold for employment may in fact be more pronounced as patients are more likely to be involved in the service sector and routine/non-technical occupations where customer or client relations are of primary importance. This speculation warrants further investigation. While “verbosity” may not be readily modifiable among clinical samples, social skills training as part of employment support in first-episode psychosis clinics may yield more robust results among patients who are on the cusp of functioning.

What does this finding mean for the study of speech, language, and communication in psychosis? As noted earlier, clinicians’ rating of disorganization tracks the deviations in grammatical encoding (connectives, pronoun use and analytic thinking index), but the social outcomes relate more with the aspects of message generation or production plan (number of words). Interestingly, a causal network exists among the variables relevant to functional/positional processing, distinct from the word count. We expect future studies to parse the large feature space of computational linguistic metrics to provide further clarity on the message production vs. grammatical encoding components in psychosis.

Our study has several strengths including the assessment of minimally treated FEP subjects, the use of objective linguistic analysis and careful control of known confounders. Nevertheless, several limitations warrant consideration. The use of a binarized NEET status has a few limitations that warrant consideration, including failing to capture “underemployment,” and its inability to capture the complexity of biological, psychological, and social factors underlying vocational outcome, and the potential instability of this metric for patients who experience a relapse of psychotic illness. Despite these limitations, the consistency between NEET status and SOFAS score (which includes a broader definition of functioning) suggests that this construct is indeed a valid measure of functioning, and simple vocational status remains a relevant goal for patients undergoing mental health treatment.

Further limitations include the lack of sufficient longitudinal speech data to assess the stability of ‘verbosity’ over time in this sample, and the lack of information on many mediators of educational/vocational success, e.g., parental support, workplace mentorship, motivational factors, or ratings of social desirability. As a result, our findings pertaining to the value of word counts in forecasting later functioning should be considered complementary information rather than being the best of all baseline predictors of functioning. Such a conservative interpretation also fits with effect-size noted in the primary Bayesian analysis (BF > 2 relating number of words to NEET status). Nevertheless, the use of acyclic graph models on longitudinal data allows us to draw causal inferences (49) from observational design. Finally, two limitations in clinical follow-up are present: the lack of longitudinal antipsychotic medication and the degree of clinical severity at the time of our follow-up vocational assessment. Antipsychotic medications may reduce articulation speed and reduce sentence length in patients with psychosis; thus patients with superior verbal output at baseline may be well positioned to offset any adverse treatment effects from antipsychotic medication, achieving superior vocational outcomes. However, in our sample, we were not able to assess the effect of long-term antipsychotic exposure on speech or clinical severity. Despite this gap, our data revealed that patients with high speech production at baseline tended to do better over time, despite no evidence of systematic differences in antipsychotic exposure or clinical severity at baseline. In future analyses, assessment of the association between baseline speech production, as well as longitudinal antipsychotic exposure and clinical response with vocational outcomes may allow researchers to parse the relationships between these variables which may provide more clarity on the mechanism underlying our observed relationship.

To conclude, we call for including the rate of word production during routine clinical assessments of first-episode psychosis. Our results suggest that this approach, while inexpensive and not requiring exhaustive training, may carry prognostic value above what is currently captured in general clinical practice.

## Data availability statement

The raw data supporting the conclusions of this article will be made available by the authors, without undue reservation.

## Ethics statement

The studies involving human participants were reviewed and approved by Western University Research Ethics Board, London, Ontario, Canada. The patients/participants provided their written informed consent to participate in this study.

## Author contributions

MM prepared the first draft, recruitment, data collection, and data analysis. RL and AS designed the analysis and implemented statistical procedures and critical review of the paper. HG, JR, and PS: contributed to recruitment and collecting the data, and critical review of the paper. LP conceived the study, designed the analysis, collected the data, and performed critical review of the paper. All authors contributed to the article and approved the submitted version.

## Funding

This study was funded by CIHR Foundation Grant (FDN 154296) to LP; AMOSO Opportunities fund to LP; Bucke Family Fund and support from the Chrysalis Foundation to LP. LP acknowledges salary support from the Tanna Schulich Chair of Neuroscience and Mental Health (Schulich School of Medicine, Western University: 2019–2022) and a Chercheur-Boursier Award from the Fonds de recherche du Québec-Santé (FRQS, 2022); program support from Monique H. Bourgeois Chair in Developmental Disorders and the Graham Boeckh Foundation (Douglas Research Centre, McGill University). We thank the Jonathon and Joshua Memorial Scholarship for their generous support of MM.

## Conflict of interest

LP receives book royalties from Oxford University Press, editorial stipend from the Canadian Medical Association Journal, and income from the SPMM MRCPsych course. LP has received investigator-initiated educational grants from Otsuka, Janssen, and Sunovion, Canada (2017) and speaker fee from Otsuka and Janssen, Canada (2019), and Canadian Psychiatric Association (2019). LP and MM received support from Boehringer Ingelheim to attend an investigator meeting in 2017. All other authors report no potential conflicts of interest. The remaining authors declare that the research was conducted in the absence of any commercial or financial relationships that could be construed as a potential conflict of interest.

## Publisher’s note

All claims expressed in this article are solely those of the authors and do not necessarily represent those of their affiliated organizations, or those of the publisher, the editors and the reviewers. Any product that may be evaluated in this article, or claim that may be made by its manufacturer, is not guaranteed or endorsed by the publisher.
